# Objective measurement of subjective tinnitus using the acoustic change complex

**DOI:** 10.1371/journal.pone.0188268

**Published:** 2017-11-27

**Authors:** Ji-Hye Han, Joong Yeon Won, Sung Kwang Hong, Ja Hee Kim, Eun Soo Kim, Hyung-Jong Kim, Hyo-Jeong Lee

**Affiliations:** 1 Laboratory of Brain & Cognitive Sciences for Convergence Medicine, Hallym University College of Medicine, Chuncheon, Republic of Korea; 2 Department of Otolaryngology, Hallym University College of Medicine, Chuncheon, Republic of Korea; 3 Department of Neuroradiology, Hallym University College of Medicine, Chuncheon, Republic of Korea; Harvard Medical School, UNITED STATES

## Abstract

At present, there is no objective method for diagnosing subjective sensorineural tinnitus. Recently, the acoustic change complex (ACC) has been used to evaluate neural detection of sounds. Thus, the present study aimed to examine whether the ACC can reflect cortical detection and discrimination of sounds matched with tinnitus frequencies. We hypothesized that the ACC to change stimuli matched with tinnitus frequencies would be decreased in tinnitus patients because the tinnitus interferes with the perception of acoustic changes. To test the hypothesis, 96 ears of normal-hearing (NH) tinnitus patients and controls were tested. Among the tinnitus patients, 33 ears with a tinnitus frequency of 8 kHz constituted the tinnitus group, and the remaining 63 ears with no experience of tinnitus were allocated to the control group. For the 4 kHz non-tinnitus matched frequency, a subset of tinnitus (n = 17) and NH (n = 47) subjects was tested. The acoustic stimuli were pure tones with a total duration of 500 ms consisting of a 1 kHz tone in the first 250 ms and a second tone of either 8 kHz or 4 kHz in the latter 250 ms. The normalized amplitude of the ACC (naACC) was calculated separately for the amplitude of the N1’-P2’ complex evoked by an 8 kHz or 4 kHz change stimulus and for the amplitude of the N1-P2 complex elicited by the initial 1 kHz background stimulus. Our results showed that the naACC to an 8 kHz stimulus in the tinnitus group was significantly smaller than those to 4 kHz and 8 kHz in normal controls. Additionally, in the tinnitus group, the naACC to 4 kHz was greater compared to 8 kHz. The receiver operating characteristic (ROC) curve analysis conducted for naACC to 8 kHz at UCL revealed a fair degree of diagnostic efficacy. Overall, our results indicated that the ACC to a change stimulus matched with the tinnitus frequency can provide an objective measure of frequency-specific tinnitus.

## Introduction

Tinnitus is the subjective perception of sound without an external acoustic stimulation. Tinnitus is a common hearing disorder, with a prevalence ranging from 10% to 20%, and approximately 40% of people with tinnitus report severe impairments in their quality of life [1–4]. Due to its subjective nature, a verification of tinnitus depends solely on a subjective report from the patient.

In animal models of tinnitus, a modification of the acoustic startle reflex (ASR) has been used as an objective measure of tinnitus [5–8]. For example, in normal rats, a silent gap inserted into background noise decreased the ASR in response to a loud startling stimulus, while in the animals with tinnitus, the background noise with a frequency close to the tinnitus frequency did not decrease the ASR [5]. To explain this lack of inhibition, it was suggested that the tinnitus sound fills the silent gap inserted into the background noise, which is close to the tinnitus frequency. Thus, animals with tinnitus perceived the stimulus as noise with no gap. This objective measure of tinnitus in animals was recently applied to tinnitus patients in an attempt to develop a clinical diagnostic tool [9]. Although a hypothesis that a tinnitus sound fills in a gap appears highly plausible, previous studies have shown that the decreased magnitude of a startle reflex in tinnitus patients was not specific to the tinnitus frequency, and the studies attributed the disinhibition of ASR to other central auditory circuits involved in gap processing. However, a psychoacoustic study paradoxically reported that tinnitus patients did not show a perceptual deficit in a gap detection task [10].

Recently, the auditory change complex (ACC) comprised of the cortical N1-P2 responses evoked by stimuli with acoustic changes has been used to assess neural detection of sounds in normal-hearing people [11,12], as well as people with cochlear implants [13]. Following the onset responses (N1-P2) to an initial acoustic stimulus, the ACC (N1’-P2’) develops when the change stimulus is presented. The ACC has been evoked by frequency and amplitude modulations and different speech tokens [11–13]. These previous studies have shown that the change in ACC as a function of various stimulus features was related to behavioral performances [11,12]. Taken together, these findings suggested that the ACC to acoustic changes could be used as an objective measurement of auditory perceptual skills.

To confirm a hypothesis suggested by previous behavioral results that a sound with a tinnitus frequency fills in silent gaps in background noise, the present study examined the change in ACC as functions of tinnitus frequency and stimulus intensity level. We hypothesized that tinnitus patients cannot clearly perceive acoustic changes in stimuli that result in decreased ACC responses. In the current study, we measured the ACC in normal-hearing (NH) subjects with tinnitus at 8 kHz using a tinnitus frequency matched stimulus (8 kHz) and an unmatched stimulus (4 kHz). We assumed that the ACC would exhibit distinctive cortical responses to a tinnitus matched stimulus in tinnitus patients because of the inhibition function for the tinnitus frequency.

## Materials and methods

### Study design and participants

In total, 96 ears (males = 34) of tinnitus patients with normal hearing were included in this study. Among these ears, 33 ears (males = 0, right handers = 19, age mean ± s.d. = 38.70 ± 14.72 years) showed tinnitus with a frequency equal to a pure tone of 8 kHz. The tinnitus patients were consecutively enrolled from our tinnitus clinic. Control subjects were recruited from the region near the hospital using a posted advertisement and community newsletter. The control subjects were not informed about a condition of the study since the gold standard of tinnitus diagnosis depends solely on self-reporting. The subjects completed self-reporting regarding otological and general medical histories, as well as a tinnitus experience. Only subjects who had never experienced tinnitus were included as controls (n = 63, males = 24, right handers = 30, mean age ± s.d. = 37.70 ± 12.24 years). All tested ears had a normal pure tone average threshold ≤ 25 dB HL at 0.5, 1, 2, and 3 kHz, and a hearing threshold ≤ 40 dB HL at all frequencies. Before measuring the ACC, an auditory brainstem response test was performed, and those showing an abnormal waveform or a delayed latency were excluded. Other exclusion criteria included a history of previous middle ear surgery, tinnitus with the vascular or muscular origin, and a history of neurological or psychological disease. The study protocol was approved by the Institutional Review Board of Hallym University Sacred Heart Hospital (Anyang, South Korea) (IRB No. 2013-I018).

### Tinnitus pitch and loudness matching

In the tinnitus matching process, all subjects underwent pure tone audiometry, and in subjects with tinnitus, a tinnitus test was applied to the tinnitus ears. The audiometry and tinnitus frequency matching were administered with a GSI 61 Audiometer (Grason-Stadler, Eden Prairie, MN, USA). The hearing thresholds for eight frequencies (0.125, 0.25, 0.5, 1, 2, 3, 4, and 8 kHz) were determined by the standard Hughson-Westlake procedure. The pulsed tones were used to match approximately with the tinnitus frequency, and the subjects were asked whether the tinnitus sound was similar to the pure tone perceived during audiometry or to a broad/narrow-band noise. For tinnitus pitch matching in tinnitus patients, different types of sounds, including a pure tone, speech noise, white noise and narrow-band noise, were presented at the 10 dB SL for each testing frequency. Among the sounds, pure tone and narrow band noise with octave frequencies ranging from 125 Hz to 12 kHz (125 Hz, 0.5, 1, 2, 3, 4, 6, 8, and 12 kHz) were presented to match the tinnitus pitch for each subject. After pulsed pairs of tone were alternatively presented, a subject was asked to choose which one most closely matched their tinnitus. These procedures were repeated several times to ensure correct matching. To avoid octave confusion, a two alternative forced choice procedure was employed with the matched tone and the octave above and below it. Only subjects who had their tinnitus at either a 4 kHz or 8 kHz pure tone participated in the current study. In addition, the most comfortable level (MCL) and uncomfortable level (UCL) were assessed with a 1 kHz tone in steps of 5 dB HL for all ears using a psychoacoustical approach [14]. In most tinnitus subjects, tinnitus loudness levels were approximately 3–5 dB SL, and the loudness levels of all subjects were below 10 dB SL.

### Stimuli and procedure

The acoustic stimulus was a 1 kHz pure tone with a 500 ms duration with the addition of the second frequency, which was either an 8 kHz or 4 kHz pure tone with a 250 ms duration. Thus, the first 250 ms was a 1 kHz tone followed by 250 ms of the change stimuli. Stimuli were generated using Adobe Audition 4 (Adobe Systems Co., San Jose, CA, USA). The stimuli were monaurally delivered with a rate of 0.3 per second via an ER-3A insert earphone. The sound intensity of the 1 kHz ongoing stimulus and 4 kHz or 8 kHz change stimulus were equal. For each subject, the stimulus was presented at either MCL or UCL measured at a 1 kHz to measure the ACC separately for two different loudness levels. The subjects were awake and supine during the recording. Two channel AgCI electrodes were placed at Cz as a reference and at A1 and A2 as the active and ground, respectively (Natus Medical Corp., San Carlos, CA, USA). Electrode impedances were maintained at less than or equal to 5 kΩ with an interelectrode impedance difference of less than 2 kΩ. A total of 50 trials were presented to subjects for each condition. The ACCs evoked by the 8 kHz change stimulus were measured in all 96 ears, while only 64 ears (17 tinnitus ears and 47 control ears) were used for the 4 kHz change stimulus since 12 subjects did not return to a clinic for 4 kHz testing. Electrophysiological recording was administered a week after tinnitus evaluation for all subjects.

### EEG recording and processing

**Fig 1:**
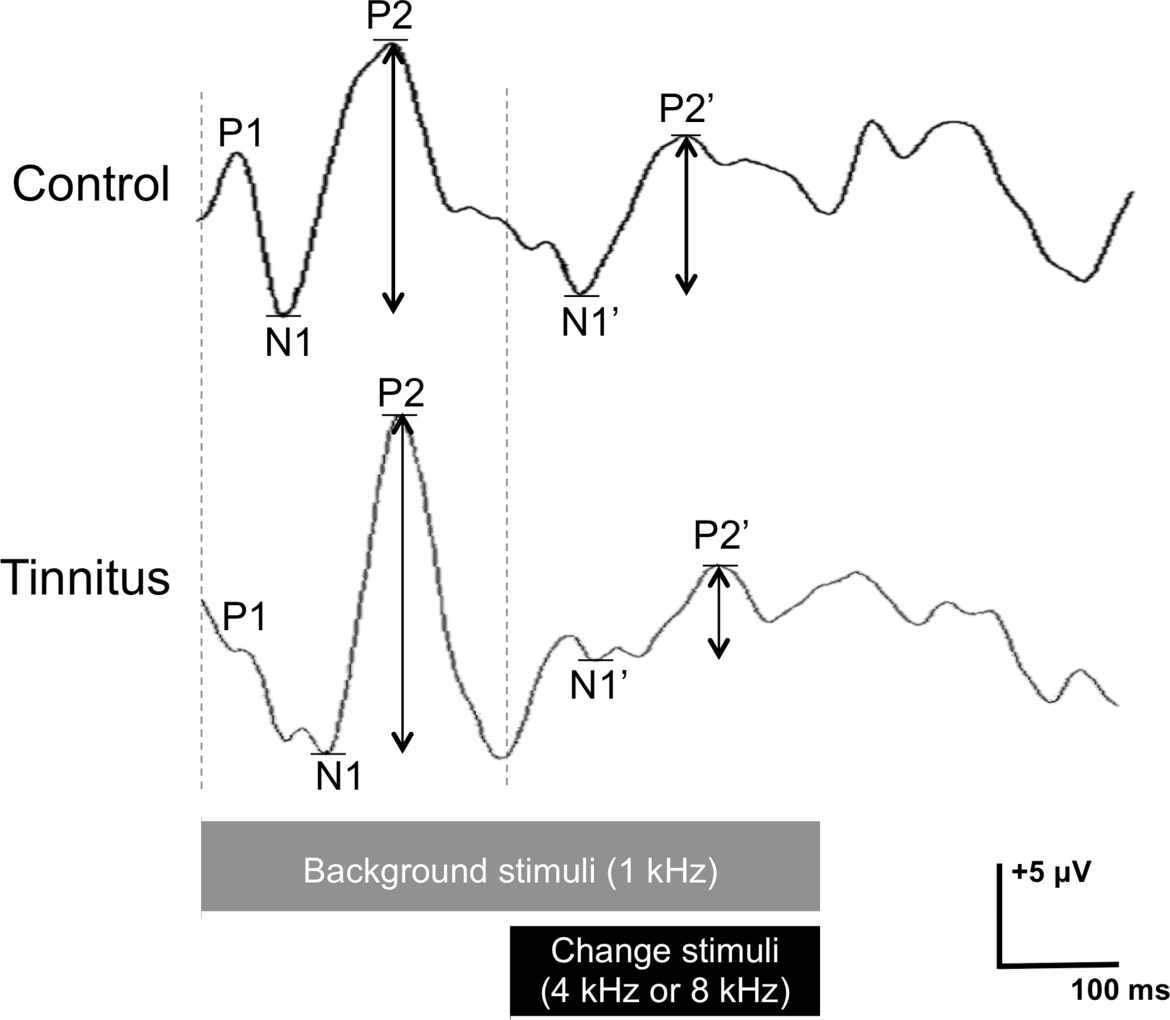
Stimulus paradigm and the representative waveforms in one tinnitus patient and a control. The stimuli were 1 kHz pure tones with a 500 ms duration in addition to either an 8 kHz or 4 kHz pure tone during the latter half. Note that the auditory change complex was evoked by the change stimuli (either an 8 kHz or 4 kHz tone), which were followed by a 1 kHz onset response.

Electrophysiological data were collected using a Bio-logic Navigator Pro System (Natus Medical Corp.) and analyzed by AEP version 7.0.0 (Natus Medical Corp.). Data were bandpass filtered (1–30 Hz) and were amplified with a gain of 30000 with an epoch duration of 500 ms. Artifacts were rejected during the test if located at 95 mV or above. The analyzer automatically picked the N1 and P2 peaks, and visual inspection of the artifact and peak detections was also administered. The N1 and P2 were defined as the most negative and positive points within a latency ranging from 80 to 200 ms for the onset response (N1-P2) and from 330 to 450 ms for the change response (N1’-P2’). The N1-P2 and N1’-P2’ were labeled used for the onset and change responses, respectively. To compute the normalized amplitude of the ACC (naACCs), the N1’-P2’ amplitudes were measured separately from the N1-P2 responses. The stimulus paradigm and the representative waveforms in a tinnitus patient and a control are depicted in Fig 1.

### Statistical analysis

A statistical analysis was performed using MedCalc Version 12.7.4.0 (MedCalc Software, Ostend, Belgium). An independent samples *t*-test was performed to assess the differences in mean age, as well as hearing thresholds at frequency regions ranging from 125 Hz to 8 kHz between the groups. Chi-square tests were used to determine whether the frequency distributions of sex and ear side differed between the groups.

For EEG recording, repeated measures analysis of variance (ANOVA) procedures were performed to examine the effects of subject group (tinnitus vs. control), stimulus frequency (4 vs. 8 kHz), and stimulus intensity level (MCL vs. UCL) on the naACC. Post hoc comparisons were conducted using Fisher’s least significant difference (LSD) test. In addition, a receiver operating characteristic (ROC) curve analysis, including all 96 ears, was performed to evaluate the diagnostic efficacy of the naACC in response to 8 kHz change stimuli presented at UCL. The criterion used for statistical significance was p < 0.05.

## Results

### Demographic data, hearing thresholds, and stimulus intensity levels

**Fig 2:**
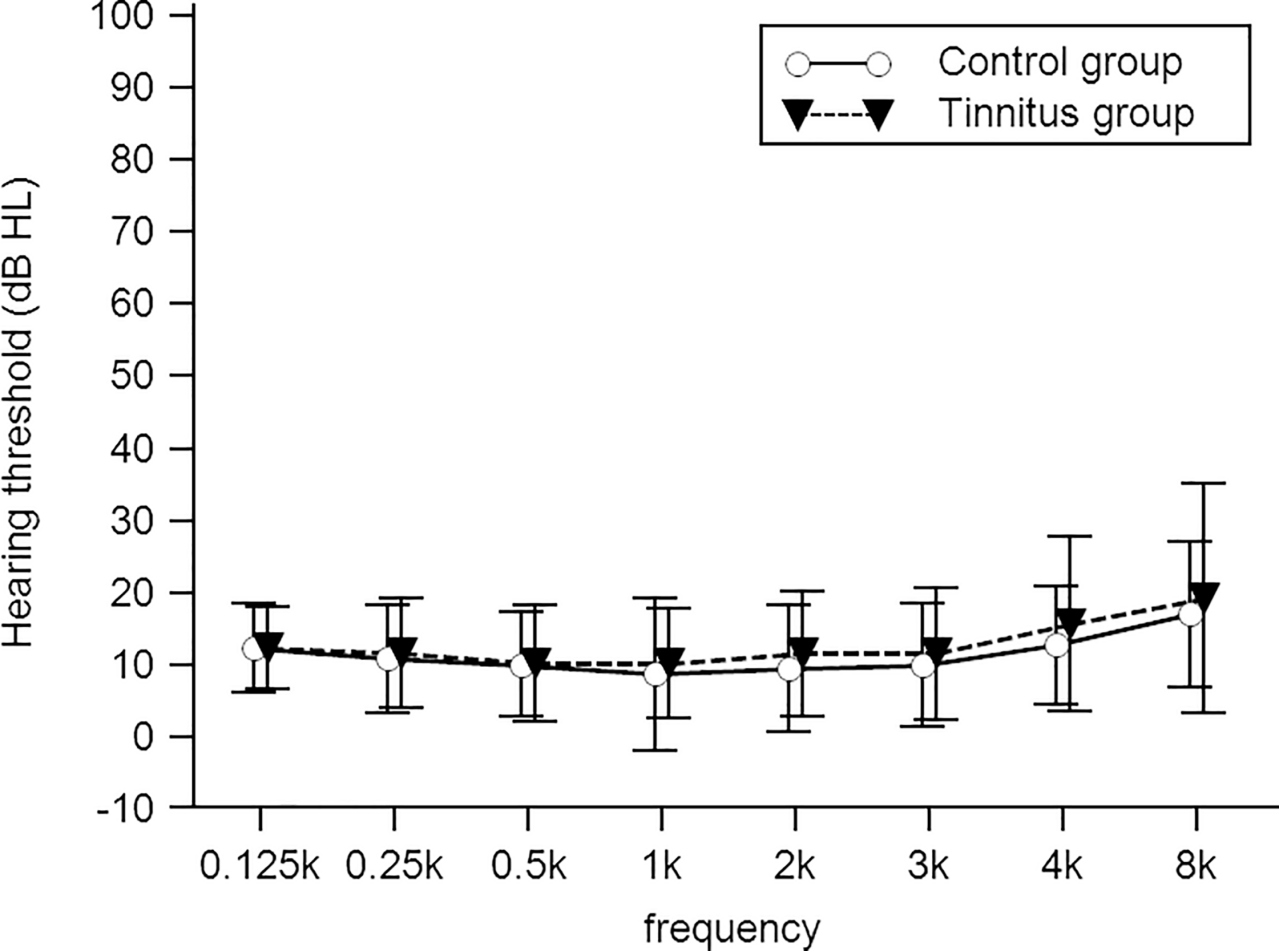
Hearing thresholds from 0.125 to 8 kHz for the tinnitus and control groups.

Demographic data showed that age, sex, and ear side were not significantly different between the tinnitus and control groups (all *p* > 0.05), (Table 1, left pane). The four-tone average threshold and hearing threshold at all frequencies tested (from 125 Hz to 8 kHz) were not significantly different between the two groups (all *p* > 0.05) (Table 1, left pane, and Fig 2). Since the stimulus intensity level was set at the MCL and UCL for a 1 kHz background stimulus, we also measured the MCL and UCL for an 8 kHz change stimulus to examine whether the loudness perception presented at the tinnitus frequency was different between tinnitus patients and controls. According to independent *t*-test results, there were no differences between the groups for both MCL and UCL measured at 8 kHz (both *p* > 0.05, Table 1, left pane).

**Table 1:** Demographic data, hearing thresholds, and stimulus intensity levels.

	All ears tested with an 8 kHz change stimulus			Ears tested with both 8k Hz and 4 kHz change stimuli		
	Tinnitus group	Control group	p-value	Tinnitus group	Control group	p-value
**number of ears**	33	63		17	47	
**gender (M:F)**	10:23	24:39	0.593	8:9	18:29	0.732
**side (R:L)**	13:20	35:28	0.197	7:10	25:22	0.571
**age (mean ± s.d)**	38.70 ± 14.72	37.70 ± 12.24	0.213	41.65 ± 15.33	39.47 ± 12.52	0.565
**Hearing threshold (dB HL)**						
**4-tone average**	10.83 ± 7.21	9.55 ± 7.56	0.425	11.47 ± 8.24	8.39 ± 6.66	0.130
**125 Hz**	12.27 ± 5.74	12.38 ± 6.15	0.933	12.06 ± 7.08	11.49 ± 5.31	0.731
**250 Hz**	11.67 ± 7.67	10.79 ± 7.42	0.590	12.06 ± 9.69	9.04 ± 6.22	0.245
**500 Hz**	10.15 ± 8.15	10.00 ± 7.24	0.926	10.29 ± 9.91	8.40 ± 5.62	0.466
**1 kHz**	10.15 ± 7.55	8.68 ± 10.54	0.479	10.00 ± 7.91	7.60 ± 11.21	0.420
**2 kHz**	11.52 ± 8.70	9.52 ± 8.74	0.291	11.76 ± 10.15	8.62 ± 7.85	0.196
**3 kHz**	11.52 ± 9.23	10.00 ± 8.61	0.426	13.82 ± 9.93	8.94 ± 7.87	0.045*
**4 kHz**	15.61 ± 12.10	12.70 ± 8.12	0.165	19.12 ± 13.83	12.34 ± 7.51	0.069
**8 kHz**	19.24 ± 15.96	17.06 ± 10.11	0.416	22.35 ± 15.72	17.23 ± 10.10	0.224
**Stimulus intensity at the frequency of change stimuli (dB SL)**						
**MCL level(at 4 kHz)**	-	-		34.41 ± 12.36	36.17 ± 6.69	0.584
**MCL level(at 8 kHz)**	31.67 ± 11.90	31.03 ± 10.05	0.783	31.18 ± 12.44	31.28 ± 10.24	0.974
**UCL level (at 4 kHz)**	-	-		60.88 ± 13.83	67.45 ± 7.44	0.078
**UCL level (at 8 kHz)**	60.76 ± 15.96	63.89 ± 9.77	0.308	57.65 ± 15.72	62.77 ± 10.10	0.224

MCL, most comfortable loudness level; UCL, uncomfortable loudness level

*p < 0.05.

### Effect of tinnitus frequency and stimulus intensity level on naACC

**Fig 3:**
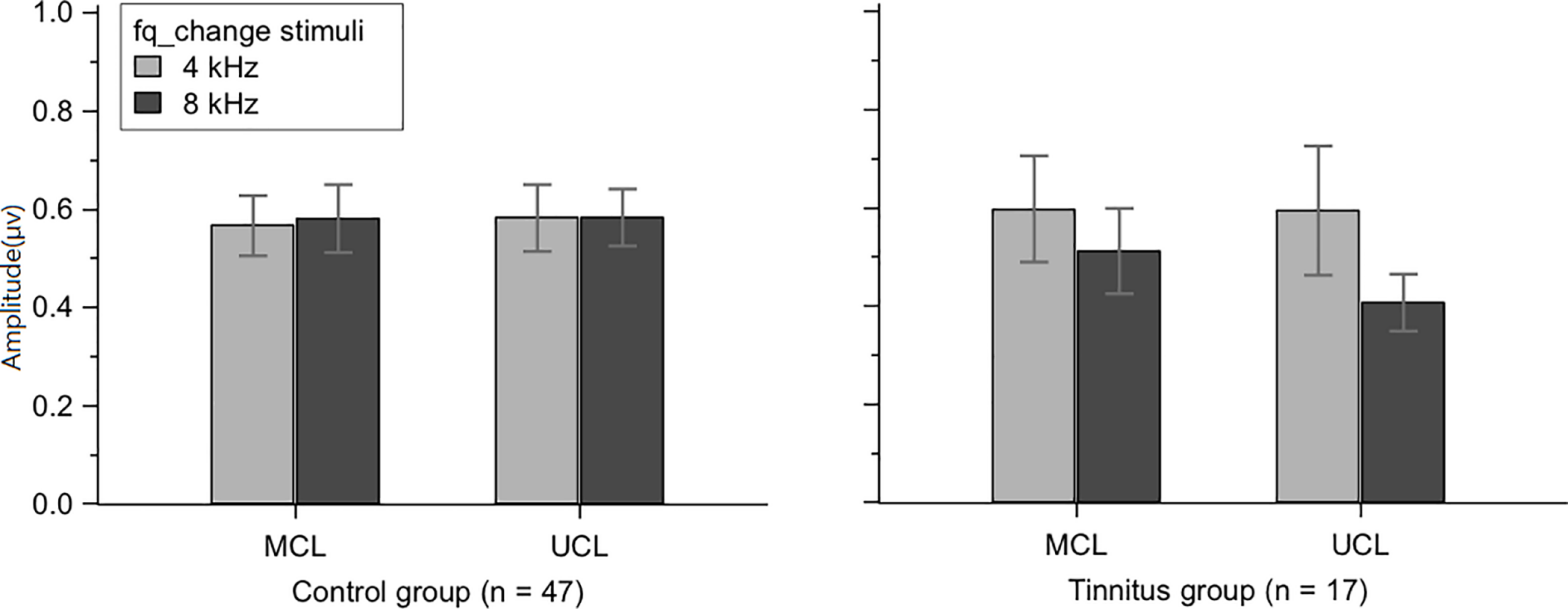
Pair-wise comparison of the naACC in ears tested with both 4 kHz and 8 kHz change stimuli in the control (n = 47) and tinnitus (n = 17) groups. The naACCs evoked by an 8 kHz frequency matched stimuli in the tinnitus group were significantly decreased compared to 4 kHz non-frequency matched stimuli in the tinnitus group as well as the control group for all conditions.

Fig 3 shows a comparison of naACCs to 4 kHz and 8 kHz change stimuli at the MCL and UCL for the tinnitus and control groups. The general trend was that in the control group, the naACCs were almost identical between 4 kHz and 8 kHz, regardless of the stimulus intensity level, while the naACCs were decreased with 8 kHz change stimuli for both MCL and UCL in the tinnitus group. Table 2 summarizes the naACC to 4 kHz (n = 17) and 8 kHz (n = 47) change stimuli for ears tested with both stimuli. A repeated measures ANOVA on the naACC (tinnitus/control x 4 kHz/8 kHz x MCL/UCL) revealed a significant group x stimulus frequency interaction [*F*(1, 62) = 4.74, *p* < 0.05]. A posthoc test showed that in the tinnitus group, the naACC to 8 kHz was smaller than those to 4 kHz (*p* < 0.05). Additionally, the naACCs to both 4 kHz and 8 kHz in the control group were greater compared to 8 kHz in the tinnitus group (both *p* < 0.05). No differences were observed between 4 kHz and 8 kHz in the control group or between the tinnitus and control groups at 4 kHz.

**Table 2:** Normalized amplitudes of the ACC to 4 kHz and 8 kHz change stimuli for ears tested with both stimuli.

Group	Stimulation level	naACC to 4 kHz (μV±SD)	naACC to 8 kHz (μV±SD)
**Tinnitus** **(n = 17)**	MCL	0.60 ± 0.21	0.54 ± 0.17
	UCL	0.59 ± 0.26	0.39 ± 0.16
**Control** **(n = 47)**	MCL	0.57 ± 0.21	0.59 ± 0.28
	UCL	0.58 ± 0.23	0.57 ± 0.22

MCL, most comfortable loudness level; UCL, uncomfortable loudness level; naACC, normalized amplitude of auditory change complex.

**Fig 4:**
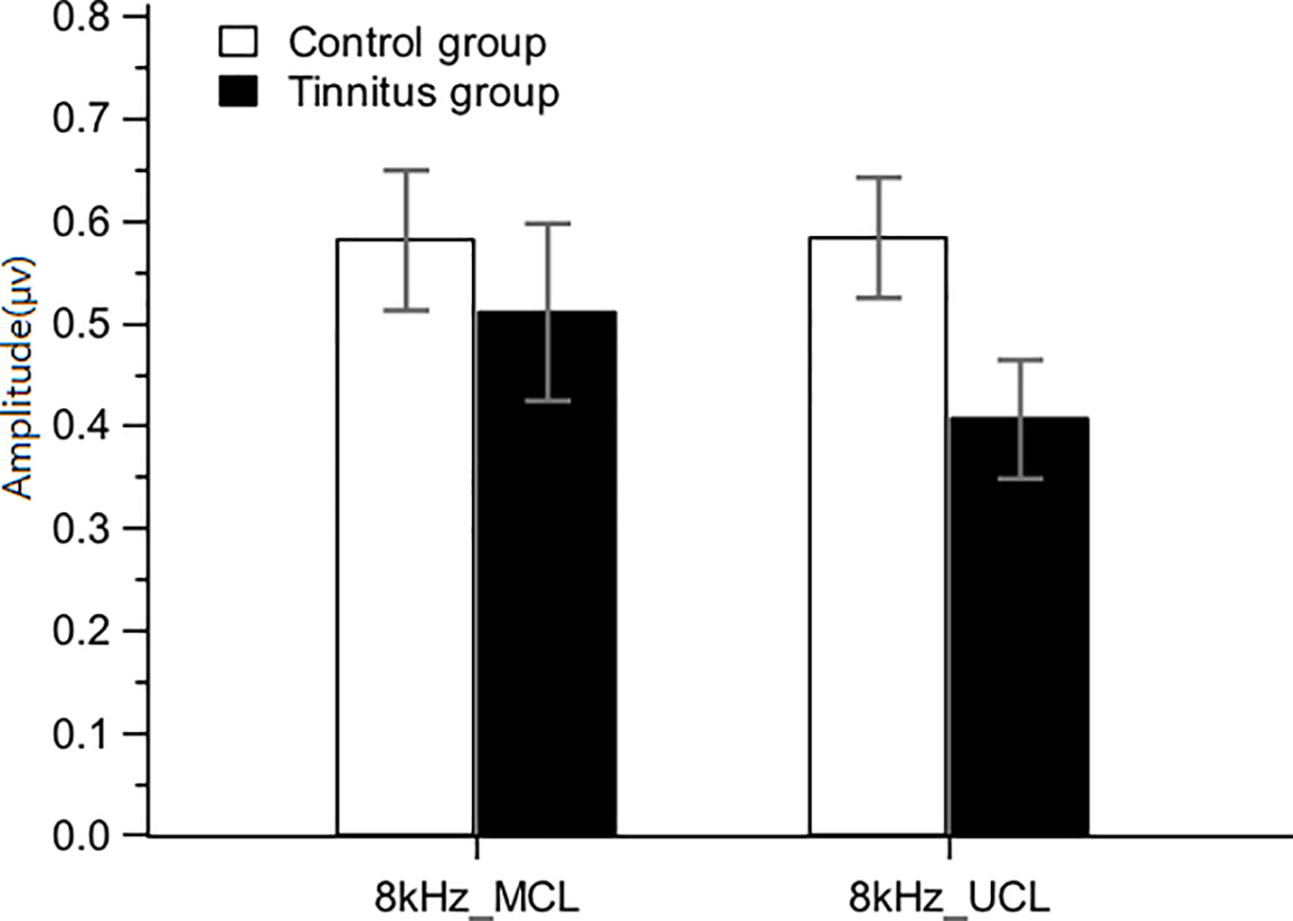
naACCs in response to 8 kHz at the MCL and UCL in the tinnitus and control groups. Note that the naACCs in the control group were significantly greater compared to the tinnitus group.

For the naACC in ears tested with only 8 kHz (n = 96), a separate statistical analysis was conducted to examine the group effect and stimulus intensity level effect. Fig 4 shows a comparison of naACCs to 8 kHz at the MCL and UCL between the tinnitus and control groups. The figure showed that the naACCs to 8 kHz in the tinnitus group were decreased compared to the control group at both MCL and UCL (Table 3). Similarly, analysis revealed a significant main effect for group [*F*(1, 94) = 9.66, *p* < 0.01], indicating that the naACCs were significantly smaller in the tinnitus group compared to the control group (*p* < 0.05). No significant difference was observed for the stimulus intensity level.

**Table 3:** Normalized amplitude of the ACC to 8 kHz change stimuli (mean ± s.d.).

	Tinnitus group (n = 33)	Control group (n = 63)
**At MCL**	0.51 ± 0.25	0.58 ± 0.27
**At UCL**	0.41 ± 0.16	0.58 ± 0.23

MCL, most comfortable loudness level; UCL, uncomfortable loudness level.

### Diagnostic efficacy of the naACC

**Fig 5:**
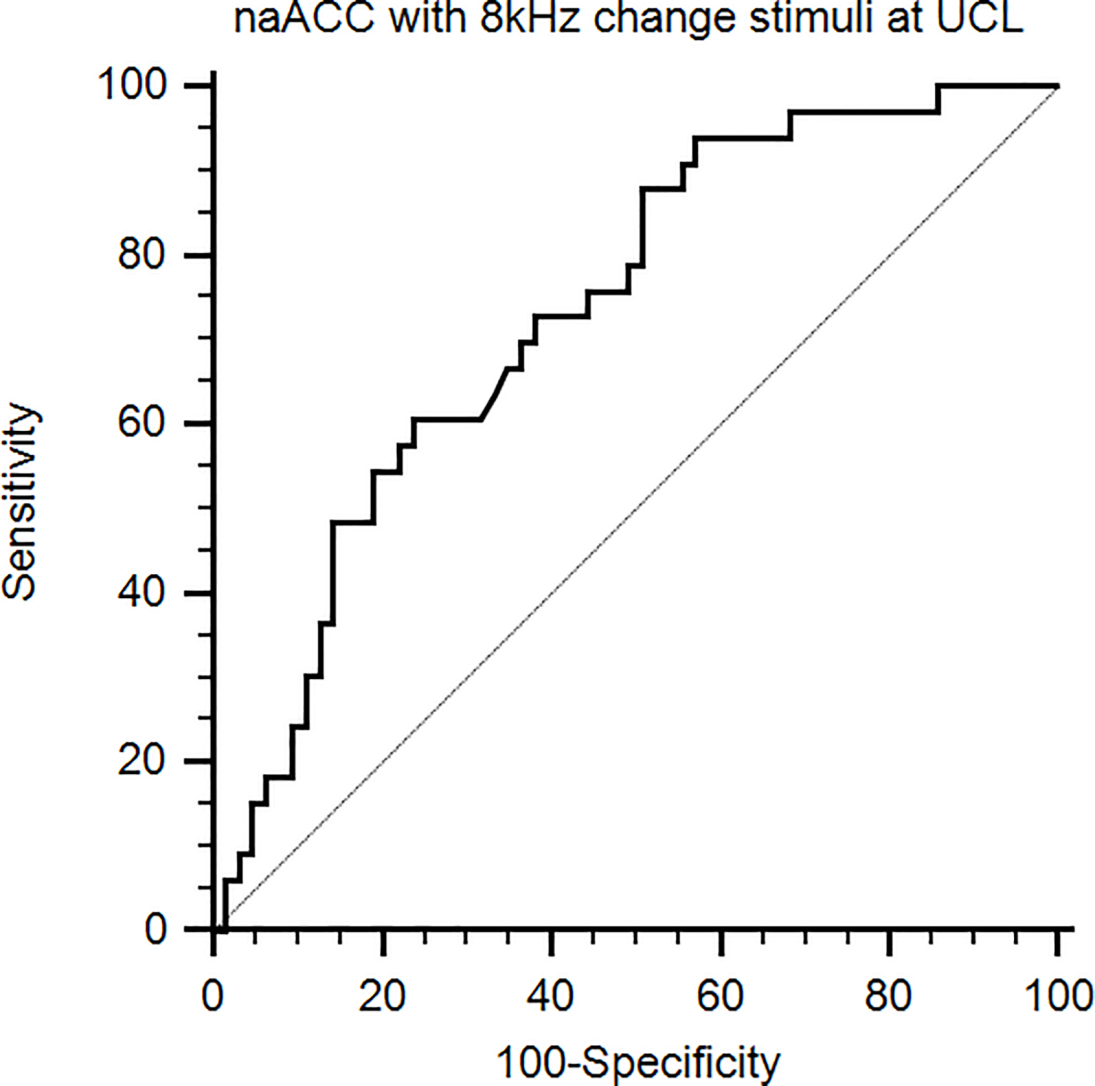
ROC curve analysis using the naACC in response to the 8 kHz change stimulus at the UCL. The area under the ROC curve was 0.732 (*p* < 0.0001).

An ROC curve analysis was performed for naACCs in response to 8 kHz at the UCL (Fig 5). The area under the ROC curve was 0.732 (95% CI: 0.632‒0.817, *p* < 0.0001), indicating a fair degree of diagnostic efficacy. When applying the suggested optimal criterion of 0.372 or less, the sensitivity was 48.48%, and the specificity was 85.71%.

## Discussion

This study aimed to describe the characteristics of the acoustic change complex in tinnitus patients to assess the potential of ACC as an objective measure for tinnitus. The naACCs were recorded using the tinnitus matched frequency at different intensity levels in tinnitus patients, as well as in normal controls. Overall, the naACCs to change stimuli were decreased in tinnitus patients compared to controls especially for the 8 kHz change stimulus. Additionally, in the tinnitus group, the naACC to 8 kHz stimulus was significantly smaller than to the 4 kHz stimulus, while no difference was found in normal controls.

In the clinic, although several audiological tests evaluating abnormalities in peripheral and central auditory organs of sensorineural tinnitus are available, an objective measure to detect subjective tinnitus does not exist. The diagnosis of tinnitus heavily relies on subjective methods, such as self-reporting, questionnaires, and visual analog rating scales. Given that the tinnitus frequency matching is associated with the frequency spectrum of hearing loss, indicating the relevance of hearing impairment to the generation of tinnitus, proper medical examination, audiological tests, and evaluation of tinnitus are required for successful management of tinnitus. However, the relationship between hearing loss and tinnitus is not straightforward in that not all patients with hearing loss have tinnitus, and a normal audiogram can be observed in people with tinnitus [1].

Previous studies have attempted to develop behavioral and electrophysiological methods for assessing tinnitus using animal models [5,15,16]. One efficient and inexpensive paradigm applied in the animal models is the gap pre-pulse inhibition of the ASR (GPIAS) [5,6,17–19]. The underlying concept of GPIAS is that tinnitus-induced animals consistently perceive the tinnitus sound in moments of silence and that the continuing tinnitus sound disturbs the perception of gaps or pre-pulses and inhibits the ASR. Although the GPIAS paradigm is widely accepted in animal studies, a recent trial in humans failed to show frequency-specific disinhibition [9].

In this study, we used a different method to identify tinnitus based on the concept of tinnitus filling in for moments of silence from the previous animal studies. We measured the change of ACC as a function of stimulus frequency and intensity level to examine whether the ACC can reflect the detection of acoustic change in tinnitus patients. The ACC is a cortical response evoked by a change in an ongoing acoustic stimulus, such as timbre, frequency, loudness, and speech sounds. The ACC has also been successfully recorded in normal hearing adults and children, as well as people with CI, to compare with their speech perception ability [11–13,20]. In tinnitus patients, a mismatch negativity study using the 8 kHz tinnitus frequency as a target stimulus showed that the N1 responses were smaller and earlier in response to the target stimuli, indicating tinnitus-induced neurophysiological changes in the tinnitus patients [21]. Furthermore, studies recording the ACC in response to frequency-specific stimuli in tinnitus patients have demonstrated that the magnitude changes in the ACC decreased when a stimulus contained the frequency corresponding to the tinnitus frequency [21,22]. The underlying theory for this finding is that if an acoustic change occurs with the frequency at which the patient hears the tinnitus sound constantly, the tinnitus sound may interfere with detection of the change.

In the present study, we found that the ACCs changed with stimulus frequency but not with stimulus intensity. Similarly, the previous electrophysiological studies examining frequency/intensity functions in tinnitus patients have reported a greater effect of frequency than the intensity. For example, Kadner and colleagues (2002) reported the intensity dependency of the N1 decreased when tinnitus frequency tones were used to evoke the N1 [22]. Weisz et al. (2005) also showed that the N1 amplitudes were increased with tinnitus-related frequencies [23]. The above findings indicated that changes of the ACC with tinnitus frequency could be due to the maladaptive plasticity of cortical tonotopic maps in tinnitus patients. The theory behind the maladaptive plasticity is that tinnitus may not be solely explained by peripheral damage, rather than by the imbalance of inhibition and exhibition of the neuronal coding. The imbalance may result from the increased activation of neurons with characteristic frequencies (CF) to the frequency range of the hearing loss. In the primary auditory cortex, neurons coding hearing loss are not able to inhibit neurons nearby, and it triggers the neurons to favorably respond to the frequency region of hearing impairment [24–26]. Consequently, the reduced inhibition of neurons with CF to the edge frequency of hearing impairment results in the phantom sensation of hearing without acoustic stimulation in tinnitus patients. A study that measured the 40 Hz steady-state response using the amplitude-modulated change paradigm also reported increased neural synchrony accompanied by tonotopic map reorganization that was similar to that shown in animal studies [27].

Another finding of our study was that in tinnitus patients, the tinnitus frequency (8 kHz) evoked significantly decreased naACCs compared to a non-tinnitus frequency (4 kHz). In addition, the naACCs to 8 kHz in tinnitus subjects were significantly smaller than those in normal controls. One possible explanation for decreased naACC amplitudes in tinnitus subjects is degraded temporal processing associated with the decreased number of phase-locked neurons for the tinnitus frequency [28]. Previous studies investigating the tinnitus-related neural change with data from cortical and subcortical recordings have reported that the decreased neural responses to stimuli with the tinnitus frequency were attributed to the poor temporal resolution, which is related to the decreased number of phase-locking neurons in the area of the auditory cortex corresponding to the tinnitus frequency [29,30].

This study has several limitations. First, the hypothesis tested in this study should be investigated with additional frequencies as well as 8 kHz for tinnitus matching. Indeed, patients sometimes fail to match their tinnitus sound to any presented sound during the frequency-matching procedure. For these patients, a participant-oriented method employed in the psychoacoustic measure may be helpful [9]. In addition, considering the diverse range of tinnitus quality, various types of change stimuli should be used to verify the potential of the ACC as a diagnostic tool. Second, although we recruited tinnitus subjects with normal audiograms at 0.5, 1, 2, and 3 kHz, a number of them (n = 14) had increased thresholds at the tinnitus frequency. It is known that sensorineural type of tinnitus is frequently accompanied by a hearing impairment, and approximately 90% of tinnitus patients have a degree of hearing loss. In addition, the remaining 10% patients could show the decreased hearing sensation at the frequencies, which are outside a typical audiogram [31,32]. Therefore, a future study should include tinnitus subjects who are free of hearing loss for all frequency ranges in the audiogram to clarify the neural correlates of tinnitus.

## Conclusions

This study determined that the ACC changed as a function of the tinnitus frequency. Additionally, a tinnitus-related neural change measured using the ACC amplitude indicated that the ACC cortical response was sensitive to subjective perception of tinnitus. Moreover, the ROC curve analysis showed a fair degree of diagnostic efficacy of the ACC. Overall, this study suggests to both clinicians and researchers that the ACC can be used as an objective measurement for tinnitus patients with a careful tinnitus frequency matching process.

## Supporting information

S1 Dataset Raw dataset of ACC for normal hearing participants and tinnitus patients. (XLS)
